# A pilot study of the associations between inflammatory markers and the presence of „deficit syndrome” in schizophrenia patients

**DOI:** 10.1192/j.eurpsy.2022.905

**Published:** 2022-09-01

**Authors:** A. Michalczyk, E. Tyburski, P. Podwalski, B. Misiak, L. Sagan, J. Samochowiec

**Affiliations:** 1 Pomeranian Medical University, Department Of Psychiatry, Szczecin, Poland; 2 Wroclaw Medical University, Department Of Psychiatry, Wrocław, Poland; 3 Pomeranian Medical University,, Department Of Neurosurgery, Szczecin, Poland

**Keywords:** deficit syndrome, Cytokines, schizophrénia, inflammatory markers

## Abstract

**Introduction:**

According to current knowledge inflammation seems to be strongly associated with pathogenesis of schizophrenia. Multiple studies and meta-analyses showed increased levels of inflammatory markers in plasma of schizophrenia patients. Individual studies have shown a relationship between the levels of inflammatory markers and the presence of deficit syndrome, but their results are inconsistent.

**Objectives:**

Analysis of associations between inflammatory markers and the presence of deficit syndrome in schizophrenia.

**Methods:**

Studied group consisted of 50 patients with diagnosed schizophrenia (F20) for at least 10 years, including 14 patients with deficit schizophrenia (DS) and 36 patients with non-deficit schizophrenia (NDS). DS and NDS did not differ significantly in age, BMI, duration of schizophrenia, types and doses of antipsychotics (chlorpromazine equivalent), but differed in sex (x2=4.28,p=0.039). Concentrations of inflammatory markers i.e. IL-6,IL-8,IL-10,TNFα,IFNγ,CRP were measured in serum using sensitive ELISA assays.

**Results:**

Initial analysis showed significantly lower concentration of IL-8 in DS compared to NDS (t=-3.18,p=0.002). This association remain significant (F=7.63,p=0.0085) after co-varying for age, sex, BMI, duration of schizophrenia, type of antipsychotic medications and antipsychotics doses. Multiple logistic regression showed that female gender (OR=0.18 [0.04-0.87],p=0.034) and higher IL-8 concentrations (OR=0.03 [0.002-0.39],p=0.007) are independent predictors of lower odds of having DS.

**Conclusions:**

Low IL-8 concentrations seem to be promising predictor of the presence of DS in schizophrenia patients, but results need further investigations. The research was funded by Polish Minister of Science and Higher Education’s program named “Regional Initiative of Excellence” in 2019–2022, grant number 002/RID/2018/2019 to the amount of 12000000PLN and by National Science Centre, Poland (2019/03/X/NZ5/00719)

**Disclosure:**

No significant relationships.

